# United States’ Emergency Department Visits for Fever by Young Children 2007–2017

**DOI:** 10.5811/westjem.2020.8.47455

**Published:** 2020-10-27

**Authors:** Sriram Ramgopal, Paul L. Aronson, Jennifer R. Marin

**Affiliations:** * Northwestern University Feinberg School of Medicine, Department of Emergency Medicine, Chicago, Illinois; † Yale School of Medicine, Departments of Pediatrics and Emergency Medicine, New Haven, Connecticut; ‡ University of Pittsburgh School of Medicine, Departments of Pediatrics and Emergency Medicine, Pittsburgh, Pennsylvania

## Abstract

**Introduction:**

Our goal in this study was to estimate rates of emergency department (ED) visits for fever by children <2 years of age, and evaluate frequencies of testing and treatment during these visits.

**Methods:**

We performed a cross-sectional study of ED encounters from 2007–2017 using the National Hospital Ambulatory Medical Care Survey, a cross-sectional, multi-stage probability sample survey of visits to nonfederal United States EDs. We included encounters with a visit reason of “fever” or recorded fever in the ED. We report demographics and management strategies in two groups: infants ≤90 days in age; and children 91 days to <2 years old. For patients 91 days to <2 years, we compared testing and treatment strategies between general and pediatric EDs using chi-squared tests.

**Results:**

Of 1.5 billion encounters over 11 years, 2.1% (95% confidence interval [CI], 1.9–2.2%) were by children <2 years old with fever. Two million encounters (95% CI, 1.7–2.4 million) were by infants ≤90 days, and 28.4 million (95% CI, 25.5–31.4 million) were by children 91 days to <2 years. Among infants ≤90 days, 27.6% (95% CI, 21.1–34.1%) had blood and 21.3% (95% CI, 13.6–29.1%) had urine cultures; 26.8% (95% CI, 20.9–32.7%) were given antibiotics, and 21.1% (95% CI, 15.3–26.9%) were admitted or transferred. Among patients 91 days to <2 years in age, 6.8% (95% CI, 5.8–7.8%) had blood and 7.7% (95% CI 6.1–9.4%) had urine cultures; 40.5% (95% CI, 40.5–40.5%) were given antibiotics, and 4.4% (95% CI, 3.5–5.3%) were admitted or transferred. Patients 91 days to <2 years who were evaluated in general EDs had higher rates of radiography (27.1% vs 15.2%; P<0.01) and antibiotic utilization (42.3% vs 34.2%; P<0.01), but lower rates of urine culture testing (6.4% vs 11.6%, p = 0.03), compared with patients evaluated in pediatric EDs.

**Conclusion:**

Approximately 180,000 patients ≤90 days old and 2.6 million patients 91 days to <2 years in age with fever present to US EDs annually. Given existing guidelines, blood and urine culture performance was low for infants ≤90 days old. For children 91 days to <2 years, rates of radiography and antibiotic use were higher in general EDs compared to pediatric EDs. These findings suggest opportunities to improve care among febrile young children in the ED.

## INTRODUCTION

Fever is the most common reason for the evaluation of pediatric patients in acute care settings.^1^,^2^ Among those evaluated in emergency departments (ED), febrile infants <90 days of age are at risk of serious bacterial infections (SBI), including urinary tract infection (UTI), bacteremia, and meningitis.^3^ Therefore, experts recommend routine testing for SBI, including blood and urine cultures, among infants <90 days with fever.^4^–^9^ In contrast, the incidence of bacteremia in children 3–36 months of age is lower, allowing for selective testing and treatment.^10^,^11^ In such patients, routine blood culture is generally not recommended.^12^ However, for febrile children older than three months of age, cross-sectional studies estimate that the overall incidence of UTI remains high (between 3–8%).^13^,^14^ Therefore, it remains important for providers to remain vigilant in evaluating for UTI. Across both age groups (<90 days and 91 days <2 years), routine use of chest radiographs is generally not recommended.^15^

Despite extensive research performed on the risk stratification of febrile children, few epidemiological data are available describing the frequency of presentation of this condition to acute care settings and rates of testing performed. Prior investigations have provided limited data with respect to infants <90 days, or have been only reported from pediatric institutions, where practice patterns may differ compared to general EDs where most children seek care.^16^–^18^ Our primary objective was to estimate the rate of ED visits for fever by infants ≤90 days, and children 91 days <2 years of age. Our secondary objective was to evaluate frequencies of blood and urine culture acquisition, radiographs, and antibiotic administration in this population and compare the management of pediatric patients 91 days <2 years between pediatric and general EDs.

## METHODS

We performed a cross-sectional analysis of the National Hospital Ambulatory Medical Care Survey (NHAMCS), a nationally representative sample survey conducted annually by the Centers for Disease Control and Prevention National Center for Health Statistics (NCHS).^19^ NHAMCS is a cross-sectional probability sample survey of ED encounters to nonfederal and short-stay hospitals in the United States. Research with NHAMCS is approved by NCHS Ethics Review Board.

We included ED encounters from 2007–2017. We evaluated two cohorts, given the disease prevalence and evidence-based management strategies: a) infants ≤90 days of age; and b) children 91 days <2 years. We identified patients with fever as those encounters with either 1) a reason for visit code (RFV) classified as “fever” (RFV 1010.0) or “feeling hot” (RFV 1012.2); or 2) a documented temperature in the ED of 100.4°F (38.0°C) or greater. NHAMCS does not document the route of temperature acquisition.

We abstracted the following: demographics; testing (including blood culture, urine culture, radiographs); antibiotics (in ED and/or prescribed); disposition; and diagnoses. We classified EDs as pediatric if >75% of encounters were by patients under 18 years of age.^20^ Results were provided using survey-weighting procedures accounting for the NHAMCS sampling design, with 95% confidence intervals (CI).^21^ We assessed presentation rates using quasibinomial regression. For patients 91 days to <2 years old, we compared rates of testing and treatment between pediatric vs non-pediatric EDs using the Rao-Scott adjusted chi-squared test. We assessed SBI rates among infants <90 days, and rates of SBI, UTI, pneumonia, and otitis media among children 91 days to <2 years in age (Supplementary Table 1).^16^,^22^,^23^ Estimates with fewer than 30 records or with a relative standard error >30% were considered unstable.^24^ We conducted analyses using the survey package^25^ in R, version 3.6.1 (R Foundation for Statistical Computing, Vienna, Austria).

**Population Health Research Capsule**

What do we already know about this issue?

*Infants ≤90 days are at risk of serious bacterial infections. In contrast, rates of bacteremia and meningitis are lower in older febrile children (91 days to 2 years)*.

What was the research question?

*Our goal was to report rates of presentation and testing among children <2 years with fever in US emergency departments*.

What was the major finding of the study?

*A lower proportion of infants ≤90 days in age are evaluated for infections. Testing in older children may be high*.

How does this improve population health?

*Findings have implication for quality improvement efforts: more testing is needed among young infants, whereas some testing among older children may be of low value*.

To evaluate specific rates of presentation and testing in 0–28, 29–60, and 61–90 day age groups, we conducted an exploratory analysis. For this, we broadened our inclusion to the years 2002–2017 in order to obtain sufficient numbers of raw patients to generate reliable estimates.

## RESULTS

An estimated 2.0 million encounters for infants ≤90 days and 28.4 million encounters for children 91 days to <2 years of age occurred over the 11 years (Supplementary Figure). Among infants ≤90 days of age, 14.8% (95% CI, 10.6–19.0%) were 0–28 days old, 41.5% (95% CI, 34.3–48.7%) were 29–60 days old, and 43.7% (95% CI, 36.6–50.9%) were 61–90 days old. There was no trend in presentation rates over time (*P* = 0.21 for ≤90 days and p = 0.10 for 91 days to <2 years).

Among patients ≤90 days, 27.6% (95% CI, 21.1–34.1%) had blood cultures, 21.3% (95% CI, 13.6–29.1%) had urine cultures, and 37.2% (95% CI, 30.4–44.0%) had radiographs (Table 1). Antibiotics were administered to 26.8% (95% CI, 20.9–32.7%). Among patients 91 days to <2 years, 6.8% (95% CI, 5.8–7.8%) had blood cultures, 7.7% (95% CI, 6.1–9.4%) had urine cultures, and 24.5% (95% CI, 22.2–26.8%) had radiographs. In this group, 40.5% (95% CI 38.5–42.5%) were given antibiotics.

Among infants 91 days to <2 years in age, encounters from general EDs had a lower proportion of urine cultures (6.4% vs 11.6%, *P* = 0.03) and a higher proportion of radiograph (27.1% vs 15.2%, *P*<0.01) and antibiotic use (42.3% vs 34.2%, *P*<0.01) compared to pediatric EDs (Table 2). Among patients 0–90 days, 9.3% (95% CI, 5.5–13.0%) were diagnosed with a SBI. Of infants 91 days to 2 years, 2.4% (95% CI, 1.8–3.0%) had a SBI (of which 91.0% [95% CI, 86.1–95.9%] had UTIs), 6.0% (95% CI, 5.1–6.8%) were diagnosed with pneumonia, and 23.4% (95% CI, 21.9–24.8%) were diagnosed with otitis media.

In our exploratory analysis for febrile infants ≤90 days old for the years 2002–2017, rates of blood cultures and urine cultures were similar between those 0–28 days and 29–60 days (Supplementary Table 2). A higher proportion of patients in older subgroups were discharged from the hospital.

## DISCUSSION

In this nationally representative sample of ED encounters, approximately 180,000 infants ≤90 days and 2.6 million children 91 days to <2 years old presented annually for fever. One-third of febrile infants ≤90 days had blood and urine cultures, while 7% of older febrile children had blood cultures. Given higher rates of bacteremia in febrile infants <90 days old, routine acquisition of blood and urine cultures is recommended by guidelines.^8^ While specific guidelines vary, all support blood and urine cultures in infants <60 days of age.^4^–^8^ While rates of bacteremia in infants 61–90 days old may be lower than rates in 0–60 days old infants, data from one recent prospective study suggest that the prevalence of bacteremia even in the third month of life is still high (1%).^26^ One study limited to pediatric hospitals found the rate of culture acquisition among febrile infants ≤90 days was 69% for blood and 75% for urine cultures.^16^

Our investigation found a low frequency of culture acquisition (27.6% having blood cultures, and 21.3% having urine cultures). However, as the rate of SBI in our study was 9% in this age group, comparable to prior research,^3^,^7^ our findings suggest a need for education and quality improvement. Quality-based measures, such as the recently reported Reducing Excessive Variability in the Infant Sepsis Evaluation, which includes clinical algorithms, order sets, education, and a mobile phone application for the management of febrile infants, can reduce variability with respect to hospital admission and lengths of stay.^27^

Bacteremia is relatively uncommon among infants >90 days of age. In one multicenter review of 57,000 blood cultures from children 3–36 months of age, rates of bacteremia were <0.5%.^11^ However, we observed that blood culture performance in this group was high (at approximately 1 in 14) and approached the rates of urine culture. Frequent use of blood cultures in this setting may lead to downstream effects, such as false positives and repeated testing.^28^ Our findings may represent adherence to older guidelines recommending empiric treatment for occult bacteremia in patients with fever. The 2003 American College of Emergency Physicians guidelines provided “Level B” evidence supporting empiric antimicrobial use for children having fever without a source.^29^ Acknowledging lower rates of bacteremia in the post-pneumococcal vaccine era, specific recommendations regarding empiric antimicrobial use were removed in a 2016 update to this guideline.^15^ Given the prevalence of viral infections in febrile children,^30^ a large number of patients may receive antibiotics unnecessarily. Educational sessions and individualized audits may be beneficial in limiting unnecessary antibiotic use.^31^

## LIMITATIONS

Our findings carry limitations, including potential errors with respect to documentation, abstraction, and coding.^32^ Some variables were not present during the entire study period. In addition, we were unable to provide reliable estimates for some tests, or obtain testing trends over time. Indications for performing particular testing and antibiotic prescribing were not available in this dataset. In particular, we were unable to directly correlate antibiotic use for specific infectious diagnoses.

## CONCLUSION

Approximately 180,000 children ≤90 days old and 2.6 million children between 91 days and <2 years present to US EDs annually with fever. Fewer than 1/3 of infants ≤90 days were evaluated with blood and urine cultures, which appears to be low. Blood culture testing and antibiotic use among children 91 days to <2 years appear to be high, in light of practice guidelines. These findings suggest important opportunities to improve the care of febrile children in the ED.

## Supplementary Information







## Figures and Tables

**Table 1:** Demographics, testing and treatment of febrile children <2 years of age.

Variable	≤90 days of age N = 2.0 million (95% CI, 1.7–2.4 million)			>90 days to <2 years N = 28.5 million (95% CI, 25.5–31.4 million)		

	Raw count*	Estimate (millions)	Estimated percent (95% CI)	Raw count*	Estimate (millions)	Estimated percent (95% CI)
Male gender	223	1.2	59.1 (51.8–66.3)	3,229	15.4	54.0 (52.2–55.8)
Race						
White	253	1.3	64.8 (57.4–72.1)	3,764	18.5	65.0 (61.6–68.4)
Black	108	0.6	27.6 (20.5–34.7)	1,735	8.1	28.6 (25.2–32.1)
Other	34	0.2	7.7 (3.2–12.2)	425	1.8	6.4 (5.2–7.5)
Ethnicity						
Hispanic	122	0.6	29.4 (22.9–35.9)	1,851	8.8	30.8 (27.4–34.2)
Non-Hispanic	273	1.4	70.6 (64.1–77.0)	4,073	19.7	69.2 (65.8–72.6)
Type of emergency department						
General	313	1.5	73.9 (65.6–82.3)	4,830	22.2	78.2 (72.5–83.8)
Pediatric	82	0.5	26.1 (17.7–34.4)	1,094	6.2	21.8 (16.2–27.5)
Seen by PA or NP without attending	50	0.2	12.2 (7.2–17.1)	877	4.7	16.5 (14.5–18.5)
Source of payment						
Private	100	0.5	23.0 (17.8–28.3)	1,306	6.4	22.6 (20.1–25.1)
Public	241	1.3	64.0 (57.6–70.4)	3,826	18.1	63.6 (60.7–66.5)
Other or not stated	54	0.3	13.0 (8.4–17.6)	792	3.9	13.8 (11.6–16.0)
Census region						
Northeast	71	0.3	13.4 (8.0–18.8)	1,115	4.0	14.1 (11.7–16.4)
Midwest	89	0.5	24.3 (16.2–32.4)	1,246	5.8	20.2 (16.4–24.0)
South	155	0.9	43.4 (35.3–51.5)	2,378	12.6	44.4 (39.1–49.7)
West	80	0.4	19.0 (13.3–24.6)	1,185	6.1	21.3 (16.7–25.9)
Cultures						
Blood culture	109	0.6	27.6 (21.1–34.1)	465	1.9	6.8 (5.8–7.8)
Urine culture†	48	0.2	21.3 (13.6–29.1)	223	1.2	7.7 (6.1–9.4)
Procedures						
Lumbar puncture†	15‡	0.1‡	6.1 (2.6–9.6)‡	9‡	0.1‡	0.5 (0.0–0.9)‡
Other diagnostic testing						
Urinalysis	179	0.8	41.4 (34.6–48.2)	908	3.9	13.8 (12.5–15.1)
Complete blood count	185	1.2	43.4 (36.2–50.7)	911	3.9	13.7 (12.1–15.2)
Radiography	139	0.8	37.2 (30.4–44.0)	1,450	7.0	24.5 (22.2–26.8)
Groups of testing†						
Blood and urine culture	37	0.2	10.5 (6.0–15.0)	58	0.3	1.2 (0.7–1.6)
Blood and urine culture with lumbar puncture	5‡	0.0‡	1.3 (0.1–2.5)‡	1‡	0.0‡	0 (0.0–0.0)‡
Therapy						
Any antibiotic	120	0.5	26.8 (20.9–32.7)	2,377	11.5	40.5 (38.5–42.5)
Disposition						
Discharged	256	1.4	66.7 (59.8–73.6)	4,908	24.1	84.7 (82.6–86.8)
Transfer	21‡	0.1‡	5.9 (2.1–9.8)‡	45	0.2	0.7 (0.4–0.9)
Admitted	77	0.3	15.2 (10.0–20.4)	259	1.1	3.7 (2.9–4.5)
Other/not stated	41	0.2	12.2 (7.0–17.5)	712	3.1	10.8 (8.9–12.8)

*CI*, confidence interval; *PA*, physician’s assistant; *NP*, nurse practitioner.

* Raw counts are the number of actual encounters available within the NHAMCS dataset; these are used with encounter-level survey weights to generate estimates and percents with confidence intervals.^21^

† Urine and lumbar puncture data were only available for years 2012–2016.

‡ Calculated from a low number of raw counts or with a high relative standard error, which may lead to estimate instability per the National Center for Health Statistics guidelines.

**Table 2:** Testing and treatment of febrile children 90 days to <2 years of age, by type of emergency department.

Variable	Pediatric emergency department visits (N = 6.2 million)	General emergency department visits (N = 22.2 million)	P-value

	Estimated percent (95% CI)	Estimated percent (95% CI)	
Seen by PA or NP without attending	15.4 (10.6–20.3)	16.8 (14.5–19.0)	0.762
Cultures			
Blood culture	8.1 (5.5–10.6)	6.5 (5.4–7.5)	0.22
Urine culture†	11.6 (6.9–16.2)	6.4 (4.6–8.3)	0.03
Other diagnostic testing			
Urinalysis	15.4 (12.3–18.4)	13.4 (12.0–14.9)	0.24
Complete blood count	11.3 (8.0–14.7)	14.3 (12.6–16.0)	0.15
Radiography	15.2 (11.4–19.0)	27.1 (24.6–29.6)	<0.01
Therapy			
Any antibiotic	34.2 (29.3–39.0)	42.3 (40.1–44.5)	<0.01
Disposition			0.10
Discharged	82.8 (77.1–88.4)	85.3 (83.2–87.4)	
Transfer	0.0 (0.0–0.1)‡	0.8 (0.5–1.2)‡	
Admitted	5.0 (3.0–7.1)	3.4 (2.5–4.3)	
Other/not stated	12.2 (7.1–17.2)	10.5 (8.5–12.5)	

*CI*, confidence interval; *PA*, physician’s assistant; *NP*, nurse practitioner.

* P-values assessed by Rao-Scott adjusted Pearson chi-squared statistic.

† Urine and lumbar puncture data were only available for years 2012–2016.

‡ Calculated from a low number of raw counts or with a high relative standard error, which may lead to estimate instability per the National Center for Health Statistics guidelines.

## References

[1] McCaig LF, Nawar EW (2006). National Hospital Ambulatory Medical Care Survey: 2004 emergency department summary. Adv Data.

[2] Li J, Monuteaux MC, Bachur RG (2018). Variation in pediatric care between academic and nonacademic US emergency departments, 1995–2010. Pediatr Emerg Care.

[3] Greenhow TL, Hung Y-Y, Herz AM (2014). The changing epidemiology of serious bacterial infections in young infants. Pediatr Infect Dis J.

[4] Dagan R, Powell KR, Hall CB (1985). Identification of infants unlikely to have serious bacterial infection although hospitalized for suspected sepsis. J Pediatr.

[5] Jaskiewicz JA, McCarthy CA, Richardson AC (1994). Febrile infants at low risk for serious bacterial infection--an appraisal of the Rochester criteria and implications for management. Febrile Infant Collaborative Study Group. Pediatrics.

[6] Aronson PL, Shabanova V, Shapiro ED (2019). A Prediction Model to Identify Febrile Infants ≤60 Days at Low Risk of Invasive Bacterial Infection. Pediatrics.

[7] Kuppermann N, Dayan PS, Levine DA (2019). A clinical prediction rule for stratifying febrile infants 60 days and younger at risk for serious bacterial infections. JAMA Pediatr.

[8] Gomez B, Mintegi S, Bressan S (2016). Validation of the “step-by-step” approach in the management of young febrile infants. Pediatrics.

[9] Greenhow TL, Hung Y-Y, Pantell RH (2016). Management and outcomes of previously healthy, full-term, febrile infants ages 7 to 90 days. Pediatrics.

[10] Stoll ML, Rubin LG (2004). Incidence of occult bacteremia among highly febrile young children in the era of the pneumococcal conjugate vaccine: a study from a children’s hospital emergency department and urgent care center. Arch Pediatr Adolesc Med.

[11] Greenhow TL, Hung YY, Herz A (2017). Bacteremia in children 3 to 36 months old after introduction of conjugated pneumococcal vaccines. Pediatrics.

[12] Rappaport DI, Cooperberg D, Fliegel J (2011). Should blood cultures be obtained in all infants 3 to 36 months presenting with significant fever?. Hosp Pediatr.

[13] Hoberman A, Chao HP, Keller DM (1993). Prevalence of urinary tract infection in febrile infants. J Pediatr.

[14] Shaikh N, Morone NE, Bost JE (2008). Prevalence of urinary tract infection in childhood: a meta-analysis. Pediatr Infect Dis J.

[15] Mace SE, Gemme SR, Valente JH (2016). Clinical policy for well-appearing infants and children younger than 2 years of age presenting to the emergency department with fever. Ann Emerg Med.

[16] Aronson PL, Thurm C, Alpern ER (2014). Variation in care of the febrile young infant <90 days in US pediatric emergency departments. Pediatrics.

[17] Gausche-Hill M, Ely M, Schmuhl P (2015). A national assessment of pediatric readiness of emergency departments. JAMA Pediatr.

[18] Hudgins JD, Monuteaux MC, Bourgeois FT (2017). Complexity and severity of pediatric patients treated at United States emergency departments. J Pediatr.

[19] Centers for Disease Control and Prevention (2019). National Center for Health Statistics: Ambulatory Health Care Data.

[20] Neuman MI, Shah SS, Shapiro DJ (2013). Emergency department management of childhood pneumonia in the United States prior to publication of national guidelines. Acad Emerg Med.

[21] National Center for Health Statistics (2015). NAMCS/NHAMCS - Estimation Procedures. https://www.cdc.gov/nchs/ahcd/ahcd_estimation_procedures.htm.

[22] Ramgopal S, Noorbakhsh KA, Pruitt CM (2020). Outcomes of young infants with hypothermia evaluated in the emergency department. J Pediatr.

[23] Ren Y, Sethi RKV, Stankovic KM (2018). Acute otitis media and associated complications in United States emergency departments. Otol Neurotol.

[24] Centers for Disease Control and Prevention (2015). NAMCS/NHAMCS - Reliability of Estimates.

[25] Lumley T (2004). Analysis of complex survey samples. J Stat Softw.

[26] Bonilla L, Gomez B, Pintos C (2019). Prevalence of bacterial infection in febrile infant 61–90 days old compared with younger infants. Pediatr Infect Dis J.

[27] Biondi EA, McCulloh R, Staggs VS (2019). Reducing Variability in the Infant Sepsis Evaluation (REVISE): A National Quality Initiative. Pediatrics.

[28] Chappell-Campbell L, Schwenk HT, Capdarest-Arest N, Schroeder AR (2018). Reporting and Categorization of Blood Culture Contaminants in Infants and Young Children: A Scoping Review. J Pediatric Infect Dis Soc.

[29] American College of Emergency Physicians Clinical Policies Committee, American College of Emergency Physicians Clinical Policies Subcommittee on Pediatric Fever (2003). Clinical policy for children younger than three years presenting to the emergency department with fever. Ann Emerg Med.

[30] Colvin JM, Muenzer JT, Jaffe DM (2012). Detection of viruses in young children with fever without an apparent source. Pediatrics.

[31] Gerber JS, Prasad PA, Fiks AG (2013). Effect of an outpatient antimicrobial stewardship intervention on broad-spectrum antibiotic prescribing by primary care pediatricians a randomized trial. JAMA.

[32] McCaig LF, Burt CW (2012). Understanding and interpreting the national hospital ambulatory medical care survey: key questions and answers. Ann Emerg Med.

