# Supplementary material for: United States’ Emergency Department Visits for Fever by Young Children 2007–2017
Source: West J Emerg Med. 2020 Oct 27;21(6):146–51. doi: 10.5811/westjem.2020.8.47455 (PMC7673886; doi:10.5811/westjem.2020.8.47455)
Supplement: Supplementary file 1 [file wjem-21-146-s001.docx]

**Supplementary Table 1.** International classification of disease (ICD) codes utilized for outcomes of serious bacterial infection and pneumonia. Diagnoses were identified if any emergency department diagnosis or hospital discharge diagnosis (for admitted encounters) had a discharge diagnosis consistent with an infection.

| **Diagnosis** | **ICD** | **Diagnosis codes** |
| --- | --- | --- |
| Urinary tract infection | 9 | 599.0, 590.10, 590.80, 599.00, 771.82, 902.0, |
|  | 10 | N10, N15.1, N12, N39.0, P39.3 |
| Bacteremia | 9 | 038.x, 771.81, 771.83, 785.52, 790.7, 995.91, 995.92 |
|  | 10 | A02.1, A41, A40, P36, R65.20, R65.21, R78.81 |
| Bacterial meningitis | 9 | 320.x |
|  | 10 | A39.0, A39.81, G00.x, G01, G04.2 |
| Pneumonia | 9 | 480.x, 481, 482, 483.x, 484.3, 485, 486, 487.0 |
|  | 10 | A37.x, J11.0, J12.x, J13, J14, J15.x, J16.x, J18.x |
| Otitis media | 9 | 381.x, 382.x, 383.x |
|  | 10 | H65.x, H66.x, H67.x, H68.x, H70.x |
