# Supplementary material for: United States’ Emergency Department Visits for Fever by Young Children 2007–2017
Source: West J Emerg Med. 2020 Oct 27;21(6):146–51. doi: 10.5811/westjem.2020.8.47455 (PMC7673886; doi:10.5811/westjem.2020.8.47455)
Supplement: Supplementary file 3 [file wjem-21-146-s002.docx]

**Supplementary Table 2**. Rates of presentation, testing, treatment and disposition among age subgroups of young febrile infants as an exploratory analysis, for the years 2002-2017.

| **Variable** | **0-28 days** | **29-60 days** | **61-90 days** |
| --- | --- | --- | --- |
|  | **Estimate** | **Estimate** | **Estimate** |
| Total presentations, millions (95% CI) | 0.57 (0.42-0.72) | 1.28 (1.02-1.54) | 1.29 (1.05-1.53) |
| Yearly estimate, millions | 0.04 | 0.08 | 0.08 |
|  | **Estimated percent (95% CI)** | **Estimated percent (95% CI)** | **Estimated percent (95% CI)** |
| **Diagnostic testing** |  |  |  |
| Blood culture | 25.0 (16.0-34.0) | 30.3 (21.5-39.2) | 30.0 (21.1-38.9) |
| Urinalysis | 36.6 (27.0-46.1) | 41.7 (33.0-50.4) | 39.7 (31.6-47.7) |
| Complete blood count | 46.1 (36.9-55.3) | 44.5 (35.4-53.6) | 43.4 (34.8-51.9) |
| Radiography | 35.1 (23.6-46.6) | 33.5 (24.3-42.8) | 42.3 (32.4-52.3) |
| **Therapy** |  |  |  |
| Any antibiotic | 33.8 (24.9-42.7) | 26.5 (19.6-33.4) | 30.5 (22.5-38.4) |
| **Disposition** |  |  |  |
| Discharged | 57.3 (47.4-67.2) | 69.8 (62.3-77.3) | 82.8 (76.8-88.8) |
| Admitted/Transfer | 36.8 (27.4-46.2) | 24.5 (17.8-31.2) | 12.1 (7.0-17.2) |
